# The Gut–Heart Axis: Effects of Intestinal Microbiome Modulation on Cardiovascular Disease—Ready for Therapeutic Interventions?

**DOI:** 10.3390/ijms252413529

**Published:** 2024-12-17

**Authors:** Alexandra Sagmeister, Christian M. Matter, Barbara E. Stähli, Michael Scharl

**Affiliations:** 1 Department of Gastroenterology and Hepatology, University Hospital Zurich, University of Zurich, 8091 Zurich, Switzerland; sagmeister.a.m@gmail.com; 2 Department of Cardiology, University Hospital Zurich, University of Zurich, 8091 Zurich, Switzerland; christian.matter@usz.ch (C.M.M.); barbara.staehli@usz.ch (B.E.S.)

**Keywords:** gut microbiome, microbiome modulation, cardiovascular disease, microbiota-derived metabolites, systematic review

## Abstract

Recent reports demonstrate an association between distinct bacteria or bacteria-derived metabolites originating from the gut microbiome and the onset or progression of cardiovascular disease (CVD). This raises the opportunity to modulate the gut microbiome to prevent or treat CVD. To investigate whether intestinal microbiome modulation can prevent or treat CVD, this systematic literature review includes all randomized clinical trials on microbiome modulation and its effects on CVD risk published between August 2018 and August 2023. Within this review, we report the modulation of the gut microbiome by a variety of interventions and their effects on CVD, focusing on cardiovascular risk factors and risk markers of CVD. Beneficial effects were observed upon lifestyle intervention and probiotics use. The most promising diets for reducing risk factors of CVD were the Mediterranean diet, high-fiber diets, polyphenol-rich diets, and diets containing polyunsaturated fatty acids. Among drug interventions, only empagliflozin showed beneficial effects on CVD risk factors. Many dietary interventions were less conclusive because of the heterogeneity of study populations, small sample sizes, and short intervention windows or follow-up. Diet, lifestyle, probiotics, or drug interventions can modulate the gut microbiome and decrease risk markers or risk factors related to CVD. Yet, their effects on clinical endpoints remain to be determined.

## 1. Introduction

Cardiovascular diseases (CVDs) are the leading cause of mortality, accounting for 32% of all deaths worldwide; a total of 17.9 million people died from CVDs in 2019 [1]. A total of 85% of all deaths by CVDs were related to coronary artery disease (CAD) or stroke [1]. In recent years, preventive medicine has made progress in reducing cardiovascular risk factors by encouraging physical activity as well as by the use of medications, such as statins, antihypertensives, and antidiabetics [2]. Nevertheless, despite all the efforts put into reducing the burden of CVDs, morbidity and mortality remain high. In addition to the established risk factors for CVDs, emerging evidence suggests that the gut microbiome may principally be involved in the pathogenesis of CVD [3,4,5].

The gut microbiota comprise all living microorganisms in the gut, including bacteria, archaea, protozoa, and fungi [6]. The microbiome includes not only microorganisms but also microbial structural elements (structural DNA/RNA, proteins/peptides, lipids, and polysaccharides), metabolites (signaling molecules, toxins, organic, and inorganic molecules), and molecules produced by the host and the environmental conditions. Even genetic elements such as phages, viruses, plasmids, prions, and virions are considered as part of the microbiome [7,8]. Numerous studies have shown a relationship between the gut microbiome and biological processes in various organ systems [9,10,11,12,13]. Moreover, recent advances in the availability of microbiome data, together with the technical progress achieved in the analysis of microbial interactions, have enhanced our understanding of the microbiome. Thus, intestinal microbiota are now increasingly considered as a target of personalized medicine to prevent and treat metabolic diseases [14].

### 1.1. Associations of the Microbiome or Microbial Metabolites with CVDs

Many recent studies published showed associations between the gut microbiome and CVD; they identified numerous pathways by which the gut microbiome could impact CVD. Recent studies have discovered several metabolites and mediators produced by the microbiome that affect the onset and/or outcome of CVD [15]. These factors are discussed more in detail as follows:

Phenylacetylglutamine (PAG) is generated from phenylalanine by the intestinal flora. PAG was shown to activate adrenergic receptors on platelets, leading to the hyperactivation of platelets and atherothrombosis, suggesting that this process could provoke major adverse cardiovascular events (MACEs) such as myocardial infarction, stroke, or death [16].

Short-chain fatty acids (SCFAs) are produced by the fermentation of indigestible dietary fibers in the colon by the gut microbiota. In a randomized controlled trial, Haghikia et al. showed the association of propionate administration with a reduction in low-density lipoprotein cholesterol (LDL-C), total cholesterol (TC), and non-high-density lipoprotein cholesterol (non-HDL-C) levels [17]. SCFAs have, furthermore, been demonstrated to have anti-inflammatory effects [18].

Many studies have examined an association between Trimethylamine N-oxide (TMAO) and CVD. Many food components, such as betaine (spinach and beetroot), L-carnitine (meat), ergothionein (mushrooms and nuts), choline, and choline-containing compounds (animal products) are metabolized to trimethylamine (TMA) by the gut microbiome [19,20]. TMAO may lead to increased platelet reactivity via a higher Ca^2+^ release and an increased thrombogenic potential [21]. A study of 4000 individuals demonstrated an association between higher TMAO levels and the risk of MACE [22].

Recent studies have demonstrated that amino acids might influence the pathogenesis of atherosclerosis. Imidazole propionate levels increase the risk of atherosclerosis and [23] are associated with increased inflammatory cytokine levels, which are associated with cardiometabolic disease progression [24]. The metabolism of bile acid is influenced by the activity of the microbiome and its concentration has been demonstrated to predict CVD [25,26,27,28,29].

Several studies have demonstrated that the microbiome or specific bacterial species thereof may trigger immune-mediated heart diseases [12]. An impaired intestinal barrier leads to the translocation of bacteria into the bloodstream and finally, inflammation. Inflammation can then trigger immune-mediated CVDs such as cardiomyopathy, atherosclerosis, and heart failure [12]. Mimic peptides from commensal bacteria can imprint cardiac myosin-specific TH17 cells and lead to a worsening of inflammatory cardiomyopathy [30]. Furthermore, the bacterial wall component, lipopolysaccharide (LPS), activates pro-inflammatory cells, and therefore, represents a risk factor for the progression of atherosclerosis [12,31].

### 1.2. Aims of the Systematic Literature Review on the Intestinal Microbiome and CVD

Most of these studies were cross-sectional or association studies and are, therefore, neither able to prove a causal effect of the gut microbiome on CVDs nor to demonstrate a therapeutic impact of microbiome modulation on CVDs in humans. Nevertheless, approaches to modulate the gut microbiome in order to prevent and treat CVDs based on pathophysiological considerations already exist. The aim of this review was to

1. Provide an overview of clinical intervention trials on gut microbiome modulation as a therapeutic target for CVDs;
2. Understand how gut microbiome modulation influences the risk of CVDs;
3. Show evidence that gut microbiome modulation prevents or treats CVDs.

**Figure 1:**
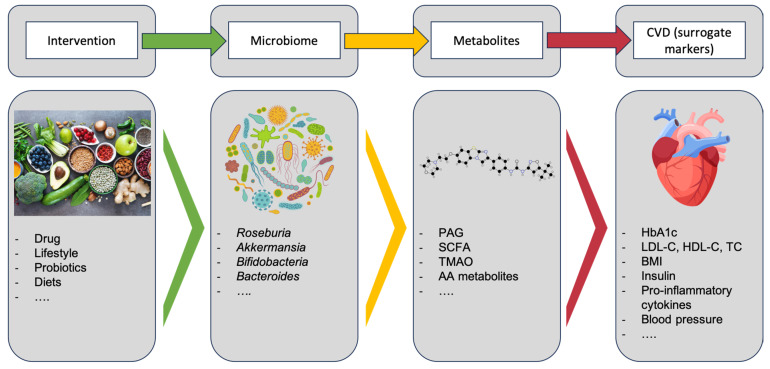
From gut microbiome intervention to modulation of metabolites and CVD risk; *PAG*: Phenylacetylglutamine; SCFA: short-chain fatty acid; TMAO: trimethylamine N-oxide; AA metabolites: amino acid metabolites; HbA1c: hemoglobin A1c; LDL-C: low-density lipoprotein-cholesterol; HDL-C: high-density lipoprotein cholesterol; TC: total cholesterol; BMI: body mass index.

Figure 1 provides an overview of the possible interventions on the gut microbiome and its metabolites to affect cardiovascular risk.

## 2. Methodology

A systematic literature analysis was conducted on PubMed, extracting articles published between 28 August 2018 and 28 August 2023 using a variety of different search terms to find all relevant articles. The following search algorithm was employed: (“gut microbiome” or “gut microbiota” or “gut microbioma”) and (“cardiovascular disease” or “cardiovascular diseases” or “atherosclerosis” or “atheromathosis” or “heart failure” or “cardiomyopathy” or “coronary heart disease”). Filters for language (English), study design (randomized, controlled trials), and publication date (5 years) were applied. The study design “randomized controlled trials” was chosen because this study design represents the highest level of evidence [32]. A total of 68 articles were identified that finally met the search criteria.

### 2.1. Inclusion and Exclusion Criteria

**Figure 2:**
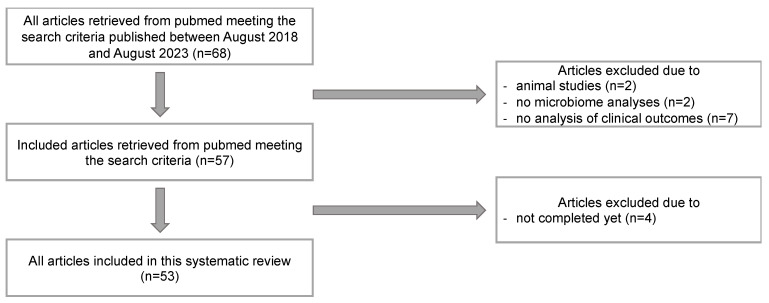
Flow diagram of literature analysis (PRISMA-based).

The studies were limited to clinical trials; therefore, studies performed on animals were excluded (n = 2). Studies were excluded if they did not include an analysis of the microbiome (n = 2) or an analysis of clinical outcomes (n = 7). Randomized controlled trials that were not completed were excluded (n = 4). Figure 2 provides an overview of the selection process applied in this literature review. The 53 remaining randomized controlled trials were included in this systematic review. The data are presented according to the PRISMA statement [33].

### 2.2. Risk of Bias

The risk of bias was assessed for each article using the Cochrane Collaboration’s tool for assessing the risk of bias in randomized trials [34].

### 2.3. Data Extraction and Analysis

The 53 randomized controlled trials were divided into the following five subgroups: lifestyle interventions, diets, probiotics, prebiotics, and drug interventions. Tables were generated to summarize the studies categorized under each of these five subgroups. For each of the studies, participant characteristics, number of participants, intervention, intervention period, effects on the gut microbiome, effects on cardiovascular endpoints, and references are listed.

## 3. Results

The following section demonstrates the effects of various interventions on the intestinal microbiome and cardiovascular endpoints. We distinguish between CV risk factors that are causally linked to an increased risk of a disease, and CV risk markers that are associated with the disease but are not causally linked.

### 3.1. Lifestyle Interventions

We first focused on the articles that studied the effects of lifestyle interventions on the intestinal microbiome and featured clinical endpoints. Five studies [35,36,37,38,39] analyzed the effects of lifestyle interventions on the intestinal microbiome and their impact on the risk of CVD (Table 1). We could include three hypocaloric diets, one endurance exercise study, and one study that differed between extensive lunches and extensive dinners. All five studies showed a significant effect on the intestinal microbiome. A significant positive clinical outcome was observed in four studies. Three studies [35,38,39] were based on a hypocaloric diet (energy-restricted diet or intermittent fasting). All these studies showed a significant positive impact on cardiovascular endpoints (e.g., a significant decrease in metabolic syndrome (MetS), LDL-C, glucose intolerance, fat mass, weight, waist circumference, and fasting glucose; significantly improved body composition, insulin sensitivity, cholesterol, and inflammatory cytokines). In parallel, significant changes in the microbiome could be demonstrated, including an increase in SCFA-producing bacteria, an increase in SCFAs, a decrease in LPS levels, and a decrease in the percentage change in TMAO. An endurance exercise study [36] also showed a significant beneficial effect on the intestinal microbiome (significant increase in *Oscillospira*; significant decrease in *Clostridium difficile*) and on clinical outcome (significant increase in VO_2_ peak and HDL-C levels; significant decrease in intrahepatic fat content and HbA1c). Of note, the timing of the main meal (extensive lunch or extensive dinner) [37] only showed an increase in *Escherichia coli* after the extensive lunch but no clinical outcomes. Four studies [35,36,38,39] found a positive correlation between changes in the intestinal microbiome and changes in CVD risk factors and markers. The results are in line with previous studies, that have also shown that an increase in SCFA-producing bacteria and SCFAs and a decrease in LPS and TMAO positively affect CVD risk factors and markers such as glucose metabolism, inflammation, atherosclerosis, blood pressure, and dyslipidemia [12,17,21,22,31,40,41,42]. To date, the effect of lifestyle interventions on the intestinal microbiome has only been demonstrated in a few studies [43]. Our review highlights that lifestyle interventions consistently result in positive effects on the microbiome. The positive clinical effect of lifestyle interventions on the risk of CVD is multifactorial and could be party explained by changes in the microbiome.

### 3.2. Diets

A total of 35 studies analyzed the effect of specific diets on the microbiome and on the risk of CVD. Given the heterogeneity among diets, a division was made into seven subgroups according to the dietary intervention. These were the Mediterranean diet, high-fiber diets, polyphenol-rich diets and polyphenol-rich phytotherapy, low- to high-fat diets, fish and meat interventions, polyunsaturated fatty acids (PUFAs), and other diets.

#### 3.2.1. Mediterranean Diet

Five studies [44,45,46,47,48] analyzed the effects of a Mediterranean diet (high in nuts, vegetables, fruits, and fish, and low in red meat) on the intestinal microbiome and the impact on the risk of CVD (Table 2). Four studies [44,45,46,47] showed significant beneficial effects on the microbiome (significant change in the abundance of different bacteria, e.g., increase in *Akkermansia muciniphila*, decrease in *Bifidobacterium*; significant increase in SCFA). A significant reduction in the risk factors and risk markers for CVD (e.g., an improved postprandial glucose metabolism and insulin sensitivity) was observed in these same articles [44,45,46,47]. In two studies *Akkermansia muciniphila* was increased; this SCFA-producing bacteria [49] has previously been shown to exert protective effects on CVD [50] (attenuating atherosclerotic lesions by improving metabolic inflammation [51], reducing plasma TMAO by increasing the urinary excretion of TMAO and TMA [52]). In addition, SCFA levels were increased in two studies [44,47] and the SCFA-producing *Prevotella* was increased in one study [45]. The study by Griffin et al. [48] comparing a healthy diet with a Mediterranean diet showed neither an effect on the microbiome nor a change in CVD risk after the Mediterranean diet. Four studies [44,45,46,47] demonstrated a positive correlation between intestinal microbiome modulation and a reduction in biomarkers related to CVD risk. A positive effect of a Mediterranean diet on risk reduction is already known [2,53]. Our review confirmed the effect of the Mediterranean diet on positive changes in the microbiome and CVD risk factor reduction.

#### 3.2.2. High-Fiber Diet

Seven studies [54,55,56,57,58,59,60] analyzed the effects of high-fiber diets (whole grain, oat porridge, flaxseed, etc.) on the intestinal microbiome and the effect on the risk of CVD (Table 2). Six studies [55,56,57,58,59,60] showed a significant change in the microbiome, including an increase in SCFA-producing bacteria (e.g., *Akkermansia*, *Ruminococcaceae*, *Lactobacillus*, and *Bifidobacterium*) and SCFAs (serum acetic acid, propionic acid, and valeric acid). A beneficial outcome regarding CV risk factors and risk markers (e.g., a significant decrease in serum TC, LDL-C, non-HDL-C, and insulin resistance) was observed in four studies [55,56,58,59]. Three studies [55,56,58] showed a positive correlation between the intestinal microbiome and reduction in cardiovascular risk factors and markers. Dietary fibers provide a substrate for the gut microbiota, and thus can influence the microbiome for positive outcomes [61]. SCFAs have already been shown to have anti-inflammatory effects and a positive effect on cholesterol levels [17,18]. Based on our review, high-fiber diets may reduce risk factors for CVD by modulating the gut microbiome.

**Table 2:** Studies with Mediterranean diet and high-fiber diet. Significantly increased: ↑↑, and significantly decreased: ↓↓.

Participant Characteristics	Number of Participants (n)	Intervention	Intervention Period	Effects on Gut Microbiome	Effects on Cardiovascular Endpoints	Ref.
Overweight Italy	29	Mediterranean diet (MD) vs. control group	8 weeks	significant changes in gut microbiota; ↑↑ postprandial plasma butyric acid	↓↓ glucose and insulin responses	[44]
Obesity/Dyslipidemia Israel	294	MD vs. healthy dietary guidelines vs. Green-MD	24 weeks	MD diets: significant changes in the gut microbiome green MD: ↑↑ *Prevotella*, ↓↓ *Bifidobacterium*	MD diets: ↓↓ body weight and cardiometabolic biomarkers	[45]
Overweight Italy	82	MD vs. habitual diet	8 weeks	↑↑ fecal *Akkermansia muciniphila*	significantly ameliorate insulin sensitivity and inflammation	[46]
Overweight Italy	23	vegetarian diet (VD) vs. MD	12 weeks, crossover	significantly changed the abundance of different bacteria	MD: ↑↑ SCFAs were negatively correlated with inflammatory cytokines	[47]
Healthy probands at increased risk of colon cancer USA	115	healthy eating diet vs. MD	24 weeks	No	No	[48]
End-stage renal disease Brazil	25	amylose-resistant starch supplementation vs. placebo	4 weeks	No	No	[54]
Cardiometabolic risk France	15	chitin-glucan (CG) vs. control	3 weeks, crossover	↓↓ *Actinobacteria* phylum and ↑↑ 3 bacterial taxa	↓↓ postprandial glycemia and triglyceridemia response	[55]
Hypercholesterolemia China	62	oat porridge vs. rice porridge (control)	6 weeks and 3 days	significant alteration in beta diversity oatmeal group: ↑↑ *Akkermansia* etc.; ↑↑ SCFAs	↓↓ serum TC, LDL-C, and non-HDL-C; ↑↑ serum total antioxidant capacity	[56]
Overweight Italy	67	aleurone vs. placebo	4 weeks	↑↑ fecal *Bifidobacterium* spp.; both groups: ↑↑ *Lactobacillus* spp.	No	[57]
Cardiometabolic risk France	39	enriched with a dietary fiber mixture vs. standard bread	8 weeks, crossover	↓↓ *Bacteroides vulgatus*, ↑↑ *Parabacteroides distasonis*, etc.	↓↓ TC, LDL-C, insulin and insulin resistance	[58]
Healthy probands USA	252	flaxseed diet (FS) vs. usual diet	6 weeks, crossover	↑↑ *Klebsiella*, *Lactobacillus*, two *Ruminiclostridium genera*, etc.	bacteria associated with CVD ↓↓	[59]
MetS risk Sweden	40	whole-grain (WG) rye diet vs. WG wheat diet	8 weeks, crossover	WG rye: ↑↑ *Bifidobacterium*; ↓↓ *Clostridium* genus	No	[60]

SCFA: short-chain fatty acid; TC: total cholesterol; LDL-C: low-density lipoprotein cholesterol; non-HDL-C: non-high-density lipoprotein cholesterol.

#### 3.2.3. Polyphenol-Rich Diet and Polyphenol-Rich Phytotherapy

Ten studies [62,63,64,65,66,67,68,69,70,71] analyzed the effects of polyphenol-rich diets (five studies) or polyphenol-rich phytotherapy (five studies) (aronia, raspberries, red wine, etc.) on the intestinal microbiome and the impact on the risk of CVD (Table 3). Eight studies [62,63,65,66,67,68,69,70] showed a significant change in the microbiome (reduction in TMAO and plasma LPS; increase in SCFA-producing bacteria and microbial diversity). These modulations of the intestinal microbiome have previously been shown to have a beneficial effect on the risk of CVD [12,17,21,22,31,40,41,42] (via anti-inflammatory effects and reduced thrombogenic potential). A beneficial outcome concerning the risk of CVD was observed in seven articles [62,63,65,66,68,69,70] (risk factors such as significantly reduced plasma TC and LDL-C, blood pressure, and fasting blood glucose). The study with red wine [67] showed an effect on the intestinal microbiome (a significant remodeling of the gut microbiota) but failed to demonstrate beneficial effects on CV risk (no change in CV risk associated with TMAO concentrations). Phytotherapeutic studies with trans-resveratrol [64] and flavanols [71], both rich in polyphenols, neither demonstrated significant effects on the microbiome nor on clinical outcomes. Five studies [63,65,66,69,70] showed a positive correlation between microbiome modulation and a reduction in biomarkers related to CVD risk. The effect of polyphenol-rich foods to alter the intestinal microbiome composition has been demonstrated previously [72]. A polyphenol-rich diet has demonstrated cardioprotective effects in a previous trial [73]. Our review demonstrates a beneficial effect of a polyphenol-rich diet/phytotherapy on the microbiome and risk of CVD (risk factors).

#### 3.2.4. Low- or High-Fat Diet

Two studies [74,75] analyzed the effects of low- versus high-fat diets on the intestinal microbiome and the impact on the risk of CVD (Table 4). Both studies investigating a low-fat diet group showed positive changes in CVD risk factors and markers (significantly decreased LDL-C/HDL-C ratio, non-HDL-C, TC, LDL-C, and waist circumference) but only one study [75] demonstrated a significant effect on the intestinal microbiome (increase in *Blautia* and *Faecalibacterium*, both SCFA-producing bacteria). The positive effects on CVD risk parameters could be partly explained by the increase in SCFA-producing bacteria [76,77]. The Atkins diet [74] (high-fat diet) showed effects on the microbiome (significantly increased *Actinobacteria*) and on CV risk factors and markers. Increased *Actinobacteria* led to significantly decreased serum LDL-C/HDL-C ratio and non-HDL-C levels. Wan et al. [75] included an intervention group with a high-fat diet and demonstrated that in addition to the positive effects in the low-fat group, the high-fat group had negative effects on the microbiome and on clinical outcomes (hs-CRP). The high-fat diet led to an increase in *Alistipes* and to a decrease in *Faecalibacterium* and *Blautia*. *Alistipes* has already been associated with several cardiovascular risk factors and CVDs such as hypertension and atherosclerosis in previous studies [3]. The decrease in *Faecalibacterium* and *Blautia*, both SCFA-producing bacteria, and a decrease in SCFA may also partly explain the negative influence on CVDs [17,42]. The negative effect of a high-fat diet on the microbiome by reducing the diversity and richness of the gut microbiome compared to a low-fat diet has already been demonstrated [78,79]. In our review, the effects of low- or high-fat diets on the microbiome and CVD risk were inconsistent.

#### 3.2.5. Fish or Meat Intervention

Two studies [80,81] analyzed the effects of fish and meat interventions on the intestinal microbiome and the impact on CV risk factors (Table 4). Both studies showed a significant change in the intestinal microbiome. Wang et al. [80] compared non-meat protein sources, red meat, and white meat, and demonstrated that red meat consumption may be associated with a higher risk of CVD (due to elevated TMAO concentrations in the red meat group). Schmedes et al. [81] compared lean-seafood diet and non-seafood diet. The study showed an association between different gut bacteria and CV risk factors. Specific gut bacteria were associated with circulating TMAO levels: lower *Clostridium* cluster IV in the non-seafood diet was associated with lower TMAO levels and therefore, might be associated with lower risk of CVD. Specific gut bacteria were significantly associated with circulating triacylglycerol (TAG) and TC/HDL-C. For example, *Roseburia*, *Sutterella*, and *Erysipelotrichaceae* significantly and positively correlated with the TC/HDL-C ratio. Based on the two heterogeneous studies in this dietary group, no definitive statement is feasible about the effects of fish or meat interventions on the microbiome and CVD risk.

#### 3.2.6. Polyunsaturated Fatty Acids (PUFAs)

Three studies [82,83,84] analyzed the effects of polyunsaturated fatty acids (PUFAs) (walnuts, margarine, etc.) on the intestinal microbiome and the impact on the risk of CVD (Table 4). All three studies showed a beneficial effect on CV risk factors, but interestingly, only the diet groups with polyunsaturated fats [82,84] but not the capsule [83] altered the intestinal microbiome. Telle-Hansen et al. [82] investigated the effect of margarine (rich in PUFA) and showed a significant change in the microbiome with a significant increase in SCFA-producing bacteria (*Lachnospiraceae* and *Bifidobacterium*) and butyrate. The study demonstrated a significant reduction in a CV risk factor (TC). The study by Vetrani et al. [69] discussed a diet containing long-chain n-3-polyunsaturated fatty acids in the polyphenol-rich diet group. This intervention group showed a similar increase in *Bifidobacteria*, but did not demonstrate a change in CV risk factors. Tindall et al. [84] investigated the effect of walnuts (rich in PUFA) on the intestinal microbiome and the impact on the risk of CVD. The study showed a significant alteration in the microbiome and beneficial effects on CV risk factors (more *Lachnospiraceae* correlated with lower blood pressure and lipid levels (TC and non-HDL-C); more *Eubacterium eligens* was associated with lower brachial MAP, blood pressure, and central MAP). Storm-Larsen et al. [83] analyzed the effect of an omega-3 PUFA capsule intervention [83] and demonstrated a significant positive clinical outcome (significant decrease in LDL-C, TC, and TAG) but no alteration in the microbiome. The two studies with an intestinal microbiome modulation demonstrated a positive correlation between bacterial changes and improvement of risk factors for CVD risk after polyunsaturated fatty acid intervention. A correlation between omega-3 fatty acids and beneficial changes in the gut microbiota has already been published [85,86]. In addition, the effectiveness of replacing saturated fatty acids with PUFA in reducing cholesterol levels [87] and thus the risk of CVD [88] has already been demonstrated. Taken together, PUFAs may reduce the risk of CVD by modulating the gut microbiome.

**Table 4:** Studies with low- or high-fat diets, diets with fish or meat intervention, and diets with polyunsaturated fatty acids. Increased: ↑, significantly increased: ↑↑, and significantly decreased: ↓↓.

Participant Characteristics	Number of Participants (n)	Intervention	Intervention Period	Effects on Gut Microbiome	Effects on Cardiovascular Endpoints	Ref.
Obesity Iran	24	Atkins diet vs. low-fat diet	6 weeks, crossover	Atkins diet: ↑↑ gut *Actinobacteria* residency and serum total antioxidant capacity	both groups: ↓↓ weight, BMI, weight circumference, and non-HDL-C low-fat diet: ↓↓ LDL-C	[74]
Healthy probands China	217	lower-fat diet vs. moderate-fat diet vs. higher-fat diet	24 weeks	different bacteria higher-fat diet: ↓↓ SCFA concentration	higher-fat diet: ↑↑ hs-CRP; lower-fat diet: ↓↓ waist circumference, TC, HDL-C, LDL-C, and non-HDL-C	[75]
Healthy probands USA	113	non-meat protein vs. red meat vs. white meat	4 weeks, crossover	red meat: ↑↑ plasma and urine TMAO	assumption of a positive association between red meat consumption and ↑ CVD risk [89]; ↑↑ TMAO concentrations in red meat group	[80]
Healthy probands Denmark	19	lean-seafood diet vs. non-seafood diet	4 weeks, crossover	non-seafood diet: ↓↓ *Clostridium* cluster IV, ↑↑ *Firmicutes/Bacteroidetes* ratio	↓↓ CVD risk associated with ↓↓ Clostridium cluster IV circulating TAG and TC/HDL-C were significantly associated with gut bacteria	[81]
Healthy probands Norway	17	polyunsaturated fatty acid (PUFA) products vs. saturated fatty acid (SFA) products	3 days, crossover	PUFA: ↑↑ *Lachnospiraceae* and *Bifidobacterium* spp., ↑↑ butyrate	PUFA: ↓↓ TC	[82]
Familial hypercholesterolemia Norway	15 (144 healthy controls)	omega-3 PUFA capsule vs. placebo	12 weeks, crossover	No	omega-3 PUFA: ↓↓ LDL-C, TC, and TAG	[83]
Cardiovascular risk USA	42	walnut diet (WD) vs. walnut fatty acid-matched diet without walnuts (WFMD) vs. oleic acid replaces alpha-linolenic acid diet without walnuts (ORAD)	6 weeks, crossover	WD and WFMD: ↑↑ *Roseburia*, *Eubacterium eligens* group etc.	WD: different bacteria correlated with CVD risk parameters	[84]

Non-HDL-C: non-high-density lipoprotein cholesterol; LDL-C: low-density lipoprotein cholesterol; SCFA: short-chain fatty acid; TC: total cholesterol; HDL-C: high-density lipoprotein; TMAO: trimethylamine N-oxide; TAG: triacylglycerol.

#### 3.2.7. Other Diets

The last subgroup is a heterogeneous group of six studies [90,91,92,93,94,95] analyzing the effect of different diets on the intestinal microbiome and the effect on CVD risk factors and markers (Table 5). Ben-Yacov et al. [90] investigated the effect of a personalized postprandial targeting diet (PPT) and showed a change in the intestinal microbiome (significant increase in alpha diversity) for the PPT. A causal analysis revealed nine species mediating an association between dietary changes and clinical outcomes and three species mediating an association between PPT adherence and HbA1c, HDL-C, and TAG. The study aims to support the role of the intestinal microbiome in influencing the effects of dietary modifications on cardiometabolic outcomes.

A whole egg intervention [92] did not show an effect on the gut microbiome composition nor on the CVD-associated clinical risk. Two diets, a lacto-ovo vegetarian diet (LOV) [91] and a multifunctional diet (MFD) [93], both showed a change in the microbiome (changes in bacteria) and a significant beneficial effect on CVD risk factors and markers (e.g., significantly reduced TC and LDL-C).

Two studies [94,95] analyzed the effects of dairy products on the intestinal microbiome and the effect on the risk of CVD. Burton et al. [94] showed a significant positive effect on the microbiome for the group with fermented dairy products (yogurt and cheese) (significant reduction in postprandial TMAO responses in urine and plasma). The intervention group with non-fermented dairy products did not show a change in the microbiome (no change in the TMAO levels). Fermented dairy products led to a significantly lower risk of CVD associated with TMAO concentrations. Vors et al. [95] did not show an effect on the microbiome in the group with milk polar lipids but demonstrated a significant positive impact on CV risk factors. Both studies had small sample sizes (23 participants and 58 participants) and short invention periods (up to 4 weeks). These diet groups were heterogeneous and most had small sample sizes and short intervention and follow-up periods. Therefore, it is not possible to definitively state the effect of these diets on the microbiome and the CVD risk.

### 3.3. Probiotics

Probiotics are live microorganisms that are intended to provide health benefits by improving or restoring the gut microbiota [96]. There is a high interest in whether such bacteria might be able to exert beneficial effects on CVD. Eight studies [97,98,99,100,101,102,103,104] investigated the impact of probiotics on the composition of the intestinal microbiome and the risk of CVD (Table 6). The studies mainly applied *Lactobacilli* [97,102,103] and/or *Bifidobacteria* [98,99,100,101,103,104], (both are SCFA-producing bacteria [105,106]), together with other species. As expected, all eight studies showed an effect on the gut microbiome. The probiotic intervention led to an increase in SCFA-producing bacteria (for example, *Bifidobacterium* and *Akkermansia* spp.) and the studies partly showed a decrease in TMAO levels, which has previously been shown to have a beneficial impact on the risk of CVD [21,22]. Five probiotic studies [98,99,100,102,104] reported significant beneficial effects on CVD risk factors and markers (e.g., a significant decrease in TC, non-HDL-C, insulin, and blood pressure). In one study with different probiotic interventions [100], only the group supplemented with *Bacillus subtilis* strain DE111 showed positive effects on CVD risk factors (significant reduction in TC and non-HDL-C).

Two studies [101,103] with patients suffering from end-stage renal disease and treated with hemodialysis failed to demonstrate beneficial effects on CVD risk factors. The lack of clinical risk reduction in patients with end-stage renal disease [101,103] may be explained by the characteristics of chronic renal disease including metabolic and immune dysregulation due to uremia and an altered gut microbiome [103].

Four studies [98,99,101,104] demonstrated a positive correlation between specific intestinal microbiome changes and CVD risk improvements on a biomarker level. The positive effect of probiotics on cardiometabolic risk was already known from previous analyses [107] and was well confirmed in our study. The positive effect on CVD risk factors could be partly explained by changes in the microbiome based on our review.

### 3.4. Prebiotics

Prebiotics are food components that are intended to encourage the growth of beneficial microorganisms in the gut [96]. There was only one study that met the search criteria in the prebiotics group [108]. This trial is ongoing and was therefore excluded.

### 3.5. Drug Interventions

Five studies [109,110,111,112,113] investigated the effects of drugs (empagliflozin, rifaximin, rosuvastatin, and choline) on the microbiome composition and the risk of CVD (Table 7). Three of these studies showed a significant effect on the microbiome [109,111,113], while a significant beneficial effect on CVD risk factors and markers was observed in only one of those studies [109]. An interesting intervention with positive effects was the use of the sodium–glucose transporter (SGLT)-2 inhibitor empagliflozin [109]. Deng et al. [109] showed significant positive effects of empagliflozin on the microbiome with a significant increase in gut microbiota richness and diversity as well as increased abundances of SCFA-producing bacteria (*Roseburia*, *Eubacterium*, and *Faecalibacterium*). Importantly, they also observed beneficial effects on the CVD risk profile (significant reduction in risk factors for CVD such as HbA1c levels and blood pressure). The findings are in line with previous studies that had also shown that SCFA-producing bacteria and SCFAs have a beneficial effect on CVD risk parameters (glucose metabolism, inflammation, atherosclerosis, and the development of atherosclerosis, blood pressure, and dyslipidemia) [40,41,42]. The increase in SCFA-producing bacteria after empagliflozin intake could, thus, potentially explain the improvement in CVD risk parameters. Based on these results, the cardioprotective effects of empagliflozin are associated with an altered gut microbiome. Nevertheless, a causal role of the gut microbiome in CVD risk reduction can still not be proven.

One study demonstrated a negative effect on a risk marker related to CVD (TMAO) [113]. Here, Cho et al. [113] demonstrated that different forms of choline supplementation had a different effect on the microbiome, more specifically on TMAO production. Choline bitartrate led to significantly greater plasma TMAO changes than phosphatidylcholine and therefore, to a significantly greater risk of CVD associated with TMAO concentrations. Drug studies with rifaximin [110,111] and rosuvastatin [112] showed no significant difference in the risk of CVD between the intervention and the control group.

## 4. Summary

### 4.1. Strengths and Limitations

To the best of our knowledge, this study is the first systematic review of randomized controlled trials investigating the association between the gut microbiome and the risk of CVD. This review supports the paradigm that several microbiome interventions may beneficially affect the cardiovascular risk profile. Due to the design of this review, which only included randomized intervention studies, the review could demonstrate an association and partial correlation between microbiome modulation and a change in CV risk markers or risk factors.

**Figure 3:**
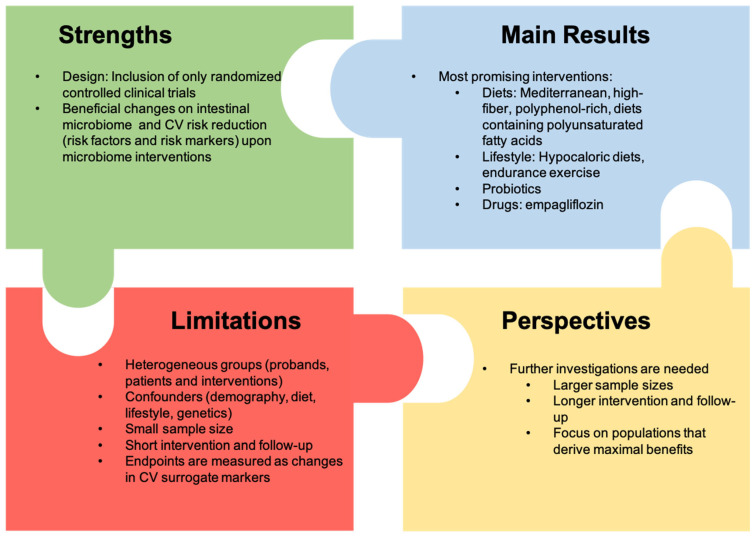
Strengths, limitations, main results, and perspectives of this study.

The study has several limitations. For a better interpretation of the study results, the studies were stratified into different intervention groups. A possible limitation resulting from this strategy is that the included participants and the interventions are heterogeneous, limiting the generalizability of the results. Furthermore, the effects of intestinal microbiome modulation are measured as changes in surrogate markers of cardiovascular risk. There is evidence that changes in risk factors translate long-term into changes in CV outcomes, but strictly speaking, these trials do not yet provide sufficient evidence for this. Despite a careful literature search using different search terms, we cannot claim that all relevant studies could be found and included. It is possible that microbiome interventions with positive outcomes were published preferentially (publication bias). Many of the studies included in this review had a small sample size, and therefore, reached low statistical power. Additionally, the intervention period and follow-up time were short in most of the studies (Figure 3).

### 4.2. Conclusions

This literature review has demonstrated the modulation of the gut microbiome by a variety of interventions. These changes in the gut microbiome were often associated and partly correlated with a decrease in cardiovascular risk markers and risk factors. Lifestyle interventions (hypocaloric diet and exercise training) and probiotics (mainly *Lactobacilli* and *Bifidobacteria*) demonstrated beneficial effects on CV risk. The most promising diets for reducing the risk of CVD were the Mediterranean diets, high-fiber diets, polyphenol-rich diets, and diets with PUFAs due to beneficial effects on risk factors and risk markers for CVDs in the majority of studies. With the exception of empagliflozin, no drug intervention trial showed beneficial effects on CV risk reduction.

### 4.3. Perspectives

Studies with longer intervention periods or follow-up times and larger sample sizes focusing on populations that derive maximal benefits are needed to clarify the effects of interventions on the gut microbiome to reduce the risk of CVD. With larger sample sizes and longer follow-up times, other factors such as demography, lifestyle, and genetics, which also influence the microbiome, might be detected.

## Figures and Tables

**Table 1:** Studies with lifestyle interventions. Significantly increased: ↑↑, and significantly decreased: ↓↓.

Participant Characteristics	Number of Participants (n)	Intervention	Intervention Period	Effects on Gut Microbiome	Effects on Cardiovascular Endpoints	Ref.
Healthy probands or metabolic syndrome (MetS) or MetS plus obesity Mexico	171	reduced-energy diet and LSFD in the first period, LSFD and functional food in the second period vs. placebo	8 weeks	↓↓ dysbiosis of gut microbiota associated with ↓↓ *Prevotella/Bacteroides* ratio	↓↓ MetS, LDL-C, small LDL particles, and LPS	[35]
Healthy probands Japan	33	endurance exercise program vs. control	5 weeks, crossover	during the exercise period: ↑↑ *Oscillospira*,↓↓ *Clostridium difficile*	during the exercise period: ↑↑ VO_2_ peak, HDL-C levels, and TC levels; ↓↓ intrahepatic fat and HbA1c	[36]
Healthy probands United Kingdom	17	large lunch vs. large dinner	1 week, crossover	after a large lunch: ↑↑ *Escherichia coli*	No	[37]
MetS China	39	intermittent fasting (IF) vs. control diet	8 weeks	↑↑ SCFA production; ↓↓ LPS	↓↓ fat mass and weight; ↑↑ improved body composition, oxidative stress, and inflammatory cytokines	[38]
Obesity USA	16	hypocaloric diet vs. eucaloric diet with exercise training	12 weeks	↓↓ percentage change in TMAO	↓↓ fasting glucose and cholesterol; both groups: ↑↑ improvement in VO_2max_, body composition, insulin sensitivity, and cholesterol	[39]

LDL-C: low-density lipoprotein cholesterol; LPS: lipopolysaccharide; HDL-C: high-density lipoprotein cholesterol; TC: total cholesterol; HbA1c: hemoglobin A1c; SCFA: short-chain fatty acid; TMAO: trimethylamine N-oxide.

**Table 3:** Studies with polyphenol-rich diet and polyphenol-rich phytotherapy. Decreased: ↓, significantly increased: ↑↑, and significantly decreased: ↓↓.

Participant Characteristics	Number of Participants (n)	Intervention	Intervention Period	Effects on Gut Microbiome	Effects on Cardiovascular Endpoints	Ref.
Overweight Switzerland	40	fruitflow (polyphenol-rich) vs. placebo	4 weeks, crossover	↓↓ *Bacteroides*, *Ruminococcus*, and *Hungatella*; ↑↑ *Alistipes*; ↓↓ TMAO and LPS	↓↓ TMAO concentrations as a marker for CVD risk	[62]
Prehypertension UK	102	aronia (polyphenol-rich) vs. Control	12 weeks	↑↑ gut microbiome richness; ↑↑ butyrate-producing species	significantly improved arterial function (augmentation index and pulse wave velocity)	[63]
Chronic kidney disease Brazil	20	trans-resveratrol vs. placebo	4 weeks, crossover	No	No	[64]
Prediabetic or healthy probands USA	26	polyphenol-dense red raspberries (RRB) vs. RRB plus fructo-oligosaccharide (FOS) prebiotic	4 weeks, crossover	RRB: ↑↑ *Eubacterium eligens* and ↓↓ *Ruminococcus gnavus*; both: ↑↑ *Bifidobacterium* and ↓↓ *Blautia wexlerae*	RRB: ↓↓ hepatic insulin resistance and plasma TC and LDL-C	[65]
High cardiovascular risk probands or healthy probands Spain	17	grape pomace seasoning (intervention group) vs. no seasoning	6 weeks	significant changes in bacteria; ↓↓ propionic acid	↓↓ blood pressure and fasting blood glucose	[66]
Coronary artery disease Brazil	42	moderate red wine consumption vs. alcohol abstention	3 weeks, crossover	significant remodeling of the gut microbiota	No	[67]
Healthy probands Italy	51	polyphenol-rich diet (PR) vs. control diet	8 weeks, crossover	*gamma-Proteobacteria* ↓↓	↓ BMI, inflammation, and dyslipidemia	[68]
High waist circumference and one or more components of MetS Italy	78	low polyunsaturated fatty acids (PUFAs) and low polyphenol (PP) vs. high PUFA vs. high PP vs. high PUFA and PP	8 weeks	PP: ↑↑ microbial diversity and *Clostridium leptum* (CLEPT); PUFA: ↑↑ *Bifidobacteria*	changes in CLEPT significantly correlated with changes in early insulin secretion	[69]
Healthy probands UK	66	aronia whole fruit vs. aronia extract vs. control	12 weeks	extract: ↑↑ Anaerostipes; whole fruit: ↑↑ *Bacteroides*	↑↑ flow-mediated dilation (FMD)	[70]
Obesity, with elevated circulating TMAO levels USA	20	four beverages with different doses of flavanols (polyphenols)	5 days, crossover	No	No	[71]

TMAO: trimethylamine N-oxide; LPS: lipopolysaccharide; TC: total cholesterol; LDL-C: low-density lipoprotein cholesterol.

**Table 5:** Studies with other diets. Significantly increased: ↑↑, and significantly decreased: ↓↓.

Participant Characteristics	Number of Participants (n)	Intervention	Intervention Period	Effects on Gut Microbiome	Effects on Cardiovascular Endpoints	Ref.
Prediabetes Israel	200	personalized postprandial-targeting diet (PPT) vs. MD	24 weeks	↑↑ alpha-diversity	detection of 3 species that mediate an association between PPT-adherence and HbA1c, HDL-C, and TAG	[90]
Healthy probands USA	19	lacto-ovo vegetarian diet (LOV) vs. LOV + cooked unprocessed lean red meat (URM) vs. LOV + cooked processed lean red meat (PRM)	3 weeks, crossover	led to changes in 23 bacteria independent of red meat intake	↓↓ serum TC and LDL-C concentrations	[91]
Overweight USA	20	2 whole eggs vs. yolk-free substitute	4 weeks, crossover	No	No	[92]
At cardiometabolic risk Sweden	47	multifunctional diet (MFD) vs. control diet	8 weeks	↑↑ *Prevotella copri*	↓↓ TC, LDL-C, LDL-C/HDL-C, ApoB/ApoA1, and TAG	[93]
Healthy probands Switzerland	Study 1: N = 13 Study 2: N = 10	Study 1: probiotic yogurt vs. acidified milk Study 2: Milk vs. cheese vs. soy drink	Study 1: 2 weeks, crossover Study 2: 1 day, crossover	fermented milk products: associated with ↓↓ postprandial TMAO responses	fermented milk products: ↓↓ CVD risk associated with TMAO concentrations	[94]
Healthy probands France	58	cream cheese devoid of milk polar lipids (PLs) vs. enriched with milk PL vs. enriched with more milk PL	4 weeks	No	milk PL: ↓↓ plasma cholesterol and TC/HDL-C, ↓↓ TAG	[95]

HbA1c: hemoglobin A1c; HDL-C: high-density lipoprotein; TAG:triacylglycerol; TC: total cholesterol; LDL-C: low-density lipoprotein cholesterol; TMAO: trimethylamine N-oxide.

**Table 6:** Studies with probiotics. Increased: ↑, significantly increased: ↑↑, and significantly decreased: ↓↓.

Participant Characteristics	Number of Participants (n)	Intervention	Intervention Period	Effects on Gut Microbiome	Effects on Cardiovascular Endpoints	Ref.
Healthy probands UK	61	cornflakes, oats, or apples all with placebo vs. cornflakes with *Lactobacillus reuteri*	8 weeks	probiotic: postprandial bile acid responses ↑↑	No	[97]
Type 2 diabetes China	365	berberine (BBR) plus probiotics (*Bifidobacterium breve*) vs. BBR plus placebo vs. probiotics plus placebo vs. placebo only	12 weeks	probiotics plus BBR: *Bifidobacterium breve* ↑↑	probiotics plus BBR was significantly superior in improving postprandial TC and LDL-C	[98]
Obesity Spain	120	enriched seafood sticks (*Bifidobacterium animalis*, inulin, and omega-3) vs. placebo	12 weeks	↑ *Lachnospiracea*, *Ruminiclostridium*, etc.	↓↓ insulin and insulin resistance; ↓↓ pulse pressure in women	[99]
Healthy probands USA	88	placebo vs. *Bifidobacterium lactis* vs. PreforPro bacteriophages plus *B. lactis* vs. *Bacillus subtilis*	4 weeks	not separately analyzed but assumed to be altered	*B. subtilis*: ↓↓ TC; improvements in endothelial function and in LDL-C	[100]
End stage renal disease China	45	probiotic (*Enterococcus faecalis*, *Bifidobacterium longum*, and *Lactobacillus acidophilus*) vs. placebo	24 weeks	significantly restored the community composition	No	[101]
Myocardial infarction (MI) Iran	44	probiotic (*Lactobacillus rhamnosus* GG (LGG)) vs. control group	12 weeks	↑↑ LGG DNA expression; ↑↑ gut microbiota’s bacterial genera levels	↓↓ serum TGF-β and hs-CRP; ↓↓ serum TMAO	[102]
End stage renal disease Brazil	21	placebo vs. probiotic (*Streptococcus thermophilus*, *Lactobacillus acidophilus*, and *Bifidobacteria longum*)	12 weeks	↑↑ betaine plasma levels	No	[103]
Obesity Spain	126	placebo vs. *Bifidobacterium animalis* (Ba8145) vs. killed form of Ba8145 (h-k Ba8145)	12 weeks	Ba8145: ↑↑ *Akkermansia* spp.	Ba8145: ↓↓ waist circumference, ↓↓ body mass index; h-k Ba8145: ↓↓ visceral fat, diastolic blood pressure, and insulin resistance	[104]

TC: total cholesterol; LDL-C: low-density lipoprotein cholesterol; TGF-β: transforming growth factor β; hs-CRP: high-sensitivity C-reactive protein; TMAO: trimethylamine N-oxide.

**Table 7:** Studies with drug interventions. Increased: ↑, significantly increased: ↑↑, and significantly decreased: ↓↓.

Participant Characteristics	Number of Participants (n)	Intervention	Intervention Period	Effects on Gut Microbiome	Effects on Cardiovascular Endpoints	Ref.
Type 2 diabetes mellitus and risk factors for CVD China	76	empagliflozin vs. metformin	12 weeks	empagliflozin: ↑↑ gut microbiota richness, ↑ SCFA-producing bacteria	↓↓ HbA1c, central obesity-related parameters, and blood pressure; ↑↑ systemic inflammatory markers	[109]
Left ventricular ejection fraction <40% and symptomatic heart failure Norway	151	rifaximin vs. *Saccharomyces boulardii* vs. control	12 weeks	No	No	[110]
Chronic kidney disease USA	38	rifaximin vs. placebo	10 days	↓↓ bacterial richness and diversity	No	[111]
Normal coronary angiograms Norway	66	rosuvastatin vs. placebo	24 weeks	No	No	[112]
Healthy probands USA	37	choline bitartrate vs. phosphatidyl-choline vs. no choline	1 day, crossover	gut microbiota composition differed between high- and low-TMAO producers	choline bitartrate: ↑↑ plasma TMAO, ↑↑ CVD risk change associated with TMAO concentrations	[113]

SCFA: short-chain fatty acid; HbA1c: hemoglobin A1c; TMAO: trimethylamine N-oxide.
